# How the perspectives of nursing assistants and frail elderly residents on their daily interaction in nursing homes affect their interaction: a qualitative study

**DOI:** 10.1186/s12877-016-0186-5

**Published:** 2016-01-14

**Authors:** Chi-chi Lung, Justina Yat Wa Liu

**Affiliations:** School of Nursing, The Hong Kong Polytechnic University, Hung Hom, Hong Kong; Centre for Gerontological Nursing, School of Nursing, The Hong Kong Polytechnic University, Hung Hom, Hong Kong

**Keywords:** Interactions, Relationships, Nursing assistants, Qualitative study

## Abstract

**Background:**

Good support from and positive relations with institutional staff can enhance the psychosocial wellbeing of residents admitted to a nursing home. Nursing assistants (NAs) interact most frequently with residents and play an important role in developing good rapport with them. Most studies have described the daily interactions between NAs and residents as task oriented. Only few have attempted to explore the perspectives of NAs and residents on their daily interactions. Therefore, the aim of this study was to identify the types of daily interactions perceived by NAs and residents. We also investigated those intentions / beliefs held by NAs and residents that might direct their interactive behaviors.

**Methods:**

A descriptive, exploratory, qualitative approach was used to explore the perspectives of 18 NAs (mean age: 51) and 15 residents (mean age: 84.4) on their daily interactions. Unstructured in-depth interviews were used to collect data. All of the interviews were conducted from July to December 2013. The collected data were transcribed verbatim and analyzed by content analysis.

**Results:**

Three types of interactions were found that described the NAs’ and residents’ perspectives on their daily interactions: (1) physiologically-oriented daily interactions; (2) cordial interactions intended to maintain a harmonious atmosphere; and (3) reciprocal social interactions intended to develop closer rapport. One or more themes reflecting the participants’ intentions or beliefs were identified from each group to support each type of interaction.

**Conclusions:**

An over-emphasis on the formal caring relationship and over-concern about maintaining a harmonious atmosphere contributed to a superficial and distant relationship between the two parties. Building close rapport takes time and involves repeated reciprocal social interactions. The findings showed that with good intentions to establish closer rapport, both NAs and residents did favors for each other. All of those favors were easily integrated in the care provided to the residents without increasing the workload of the NAs. Modifying the training given to NAs and adjusting institutional policies are crucial to raising the competence of the NAs in building good relationships with residents. Positive interactions improve the psychosocial wellbeing of the residents and encourage them to cooperate during the delivery of care, thereby improving their overall health and contributing to the NAs’ job satisfaction.

## Background

Older populations are projected to increase worldwide. The demand for long-term care services for frail older people who can no longer take care of themselves keeps rising. When admitted to a nursing home, social support from [1, 2] and positive relations with [3, 4] other residents and institutional staff have been found to contribute more to the psychosocial wellbeing of residents than support and relations from their social networks outside of the nursing home. A sense of belonging, togetherness, respect, and satisfaction with their social support were reported by those who gave a positive appraisal of their daily contacts with the nursing home staff. By contrast, feelings of being disrespected, loneliness, and social isolation were reported by residents who were dissatisfied with their daily interactions with nursing home staff [2, 5, 6]. This highlights the value of satisfactory social relationships between nursing home staff and residents.

Building rapport and providing social support both take time to achieve through daily interactions in the nursing home. Other than nursing home staff, nursing assistants (NAs) are the primary caregivers of the residents. They are the ones who interact most frequently with the residents and play an important role in their social network inside the nursing home [7]. Their interactions with the residents affect the latter’s perceptions of their psychosocial wellbeing. Many observational studies have shown that the daily communication and contact between NAs and residents are mainly task-oriented. Although sociable conversations or behaviors have been observed, these were usually described as superficial, involving no sharing of authentic personal feelings. NAs and residents were not inclined to establish closer relationships. NAs focused most of their attention on completing their tasks and had little concern for the psychosocial / emotional wellbeing of the residents [8–11].

Numerous factors can affect the interactions that take place between people, including the intentions, perceived roles, personal beliefs, and cultural norms of the individuals involved. For example, during the daily interactions between nursing home staff and residents, the former assume the role of caregiver while the latter are care recipients. They may possess similar therapeutic intentions, which may drive both parties to interact in ways that meet those intentions [12]. Interaction incorporates the concept of relationship, with people expressing themselves based on the roles that they play and influenced by self-perceptions. It also includes the concept of communication, where achieving mutual understanding between the parties involved should be a major aim [13]. How people perceive their interactions and the underlying intentions should have a significant influence on their daily interactions. However, most studies have focused on describing the daily interactive behaviors of NAs or residents rather than exploring the factors that may influence the interactive behaviors of both parties. Some have reported events that occurred in nursing homes or attempted to investigate the sentiments of residents as judged from their appraisal of their interactions with NAs. Only a few studies have explored the general perspectives of NAs and residents on their daily interactions. Those that investigated the perspectives of NAs found that NAs placed a high value on psychosocial support and on having good rapport with residents [14–16]. However, the study samples were largely limited to NAs who were experienced or educated. Also, the studies focused predominantly on interactions between NAs and residents during caring procedures [17, 18], while daily interactions also include contacts during periods of leisure. In addition, there is only limited information on how unseen individual intentions and personal beliefs affect the interactive behaviors of NAs or residents. In order to address this gap in the literature, the aim of this study was to: 1) identify the types of daily interactions perceived by NAs and residents; 2) investigate those intentions / beliefs of NAs and residents that direct their interactive behaviors.

## Methods

### Design

A descriptive, exploratory, qualitative approach was used to explore the perspectives of nursing home residents and NAs on their daily interactions. Individual, unstructured, in-depth interviews were used to collect data. This approach allows the participants to express themselves in a more natural and narrative [19], and is therefore beneficial for collecting comprehensive data on the thoughts, attitudes, beliefs, and knowledge of the participants [20].

### Setting and participants

An email was sent and a subsequent phone call describing the purpose of the study was made to non-profit nursing homes in Kowloon district in Hong Kong. Eventually, six nursing homes agreed to take part in the study. The organizational structure of a local nursing home is as follows. The superintendent is responsible for the overall operation and administration of the institution. Nurses are responsible for the overall health care of the residents in that they primarily assume managerial and supervisory roles in maintaining the standard of care. NAs are hands-on workers who render daily personal care to residents under the supervision of nurses [21]. Potential participants were nominated according to the sample selection criteria for NAs and residents. Eighteen NAs and fifteen residents were recruited by purposive sampling between July and December 2013. The sample selection criteria for participating NAs included the ability to speak Cantonese and at least 6 months of work experience in residential services. As we intended to collect opinions from NAs of different backgrounds to enhance the credibility of the findings [22], no exclusion criteria were imposed for participants in the NA group. Inclusion criteria for participating residents included those aged ≥ 65, who had been living in the nursing home for > 6 months, so as to avoid issues of anxiety and distress arising from an unfamiliar new environment, those who were cognitively intact as determined by their attainment of a score of above 19 in the Cantonese version of the Mini-Mental State Examination [23], and those who were able to communicate in Cantonese. They had to be in stable general health with no acute physiological or psychiatric illnesses, and no history of emergency hospital admissions within the past 3 months. Residents were excluded if they had severely impaired hearing and sight that might affect their ability to take part in the interview; and if they were experiencing any distressing social circumstances, for example, the recent death of a close relative. Demographic data were collected prior to each interview.

### Data collection

The study commenced after ethical approval was received from the Human Subjects Ethics Application Review System of the Hong Kong Polytechnic University. The nature and purpose of the study were fully explained to all potential participants in an information letter that they were given, and in the interview conducted with each individual immediately after they gave their informed written consent to participate in the study. All interviews were conducted by the PI (principal investigator) using an unstructured interview guide. Each interview started with broad, open-ended questions to obtain the participants’ general views, such as *“How do you perceive the daily interactions between you and the nurse assistants / residents in the nursing home?”* They were followed by more focused and individualized questions to obtain a better idea of the perspectives of the participants. Examples of such questions are given in Table 1*.* Each interview took approximately 30–45 min. All interviews followed the same interview guide to ensure the reliability of the entire study. All of the interviews were audiotaped and field notes were taken describing the setting and the PI’s general impression of the interviews.

**Table 1:** Examples of semi-structured interview questions

Opening question	
	How do you perceive the daily interactions between you and the nurse assistants / residents in the nursing home?
Probing questions	
1	In what situations do you usually interact with each other?
2	In what situations do you both usually interact with each other?
3	How do you describe the relationship between you and the residents / nurse assistants?
4	How do you perceive the interaction that you have just shared?
5	How did you feel about the interaction that you have just shared?
6	Are there any special reasons to do this to residents / nurse assistants?
7	What meaning was there in what the residents / nurse assistants did for you?

### Data analysis

All of the collected data were subjected to a qualitative content analysis [24]. All audio data were first transcribed verbatim in Chinese, checked for accuracy, and organized by the PI (interviewer) using QAR NVIVO 10.0. All of the data were analyzed independently by the PI and another coder who was familiar with qualitative research but had not conducted any of the interviews in this study. The analysis involved an iterative process of description, analysis, and interpretation, and three levels of coding. At the first level of coding, the coder/interviewer read the transcripts separately and allocated a code to each concept. In the second level, they looked for similar and redundant codes. The major theme of the coding was generated by aggregating similar codes and interpreting their interrelationships [25]. During the first and second levels of coding, all of the data collected from the NAs and residents were analyzed separately to devise an index of key concepts and coding, drawing on a priori issues linked to the aim of the study. These two sets of coding were then compared. In the third level of coding, the coder and interviewer analyzed, compared, and identified the themes from both sets of data, so as to identify relationships between and among the generated themes. By the final few interviews, it was found that all codes and themes repeated those in previous interviews. A discussion between the interviewer and the coder confirmed that the data were saturated to the point at which no new data were likely to emerge [24]. Next, the generated themes and related quotes were translated into English by the interviewer and back-translated by a translator who had not been involved in the previous coding process. The back-translation was compared with the original Chinese transcripts to ensure accurate representation of the narrative [26]. The Statistical Package for the Social Sciences (SPSS) version 21.0 was used to manage the quantitative data. Descriptive statistics were used to describe the demographic data of the participants.

### Rigor

Several strategies were used to ensure a rigorous analytical approach. To enhance the credibility of the findings, participants in both groups were selected so as to include people from a variety of backgrounds. This would help to corroborate findings related to the influence of daily interactions between residents and NAs on the residents’ psychosocial wellbeing. To encourage the participants to express deep and genuine feelings during the interview, it was emphasized to the patients prior to the interview that the confidentiality of their data and their anonymity would be maintained. In this way, the authenticity of the data could be enhanced [22]. To ensure consistency, an interview guide was developed after a comprehensive literature review of relevant topics. Five experts in the field of gerontology evaluated the cohesiveness of the lines of questioning (Table 1) with the aim of the study. Last, but not least, all levels of coding were analyzed independently and separately by the coder and the interviewer. After each level of coding had been completed, the coder and the interviewer held a discussion to identify similarities and discrepancies among the codes. When agreement between two sets of codes was high, a theme was derived. The few disagreements that arose were resolved through discussions involving the whole research team. Progress to the next level of coding was made only when an agreement was reached on the identified codes. An overall thematic framework was then developed based on the previously identified themes and their quotes. This framework was reviewed by two independent experts in gerontology, who were also involved in reviewing the interview guide, to establish the appropriateness of the themes. This method of seeking agreement between co-researchers and experts also helped to establish the credibility of the study.

## Results

All eighteen full-time NA participants were female, aged 37 to 62, with an average length of employment of 7.4 years. All of them had completed courses related to basic personal care prior to their employment in the nursing homes, and had received continuous training predominantly on the physical care of older people and the common illnesses affecting them (Table 2). The age of the fifteen resident participants ranged between 71 and 96. Their average duration of residence in the nursing homes was 5.8 years. Despite comorbidity, eight of them claimed to be able to manage most simple self-care activities such as bathing, grooming, or toileting (Table 3).

**Table 2:** Characteristics of the nurse assistant participants (*n* = 18)

Mean age (Standard deviation)		51 (±6.7)
Gender	Female	*n* = 18
Marital status	Single	*n* = 1
	Married	*n* = 13
	Divorced	*n* = 3
	Widowed	*n* = 1
Highest educational level	Primary	*n* = 1
	Secondary	*n* = 17
Religion	Buddhism	*n* = 3
	Christianity	*n* = 2
	None	*n* = 13
Length of employment	6 months - <1 year	*n* = 2
	1–5 years	*n* = 7
	6–10 years	*n* = 4
	11–20 years	*n* = 4
	>20 years	*n* = 1

**Table 3:** Characteristics of the resident participants (*n* = 15)

Mean age (Standard deviation)		84.4 (±7.3)
Gender	Female	*n* = 11
	Male	*n* = 4
Mean ^a^CMMSE score		23.9
Marital status	Single	*n* = 1
	Married	*n* = 5
	Divorced	*n* = 3
	Widowed	*n* = 6
Highest educational level	None	*n* = 1
	Primary	*n* = 9
	Secondary	*n* = 4
	Tertiary or above	*n* = 1
Religion	Buddhism	*n* = 4
	Christianity	*n* = 5
	None	*n* = 6
Duration of residence	6 months - <1 year	*n* = 2
	1–5 years	*n* = 7
	6–10 years	*n* = 3
	11–20 years	*n* = 3
No. of diagnosed conditions under treatment or observation	2 or fewer	*n* = 1
	3–5	*n* = 7
	6 or more	*n* = 7

^a^ *CMMSE* Cantonese Mini-Mental State Examination

Three types of interactions were found that describe the NAs’ and residents’ perspectives on their daily interactions in the nursing homes: (1) physiologically-oriented daily interactions; (2) cordial interactions intended to maintain a harmonious atmosphere; and (3) reciprocal social interactions intended to develop closer rapport. One or more themes reflecting the participants’ intentions or beliefs were identified from each group to support each interaction that was identified. Interestingly, some themes that were identified from the NA group were similar to or echoed those identified from the resident group, which supported the related interactions. All of the interactions, major themes, sub-themes, and related illustrative quotes that were identified are listed in Table 4.

**Table 4:** Types of interactions, major themes, sub-themes, and sample quotes

Nurse assistant group		Resident group	
Theme and sub-theme	Quotes	Theme and sub-theme	Quotes
First type: Physiologically-oriented daily interactions			
Worrying about going beyond the boundaries of an NA’s responsibilities	“‘My daily contacts with residents mainly take place during the time that we care for them … doing different nursing procedures … this is our everyday routine in the nursing home…. I am just an NA and have not received any training in handling the residents’ other problems. I am only responsible for conducting basic personal care.” (NA 4)	Boundaries inherent in the nature of an NA’s role as a formal physical caregiver	“There is not much chatting between the NAs and me because, as a resident here, and the NAs are the workers, so we lead different lives…. There is nothing to talk about … I mean, we do not interact like friends; there is not much joking, or chatting.… These things are all on me, and are none of their business…. I wouldn’t force anyone to be my close friend, I’ve never thought about that.... We, residents are those who need to be taken care of … they are care workers…. Our relationships can’t go beyond this…. I mean, there is no way for us to be friends at all.” (RE 11)
Falling under the responsibility of other professionals	“If residents are not in a good mood, or have a family problem that is affecting them, we report the cases to social workers…. We shouldn’t ask much about it because it might trigger their sadness. We are not social workers, and we are afraid of saying something wrong…. So I think it is not my job, and is something beyond my abilities. Developing a closer relationship with residents is not our major duty, based on our role in the nursing home.” (NA 3)	Driven by the residents’ demands relating to their physiological needs	“They care about our (the residents’) health, not our family background, personal issues…. They mostly care about our physiological needs … whether or not we can take care of ourselves…. They offer help when we can’t look after ourselves and do things for us.” (RE8)
			“I seldom ask for their help, as I can do things (grooming, dressing, etc.) on my own…. It is really not necessary to talk with them (NAs).” (RE7)
Perception of incompetence and irrelevance	“We wouldn’t dig deep into the residents’ personal issues. Because we wouldn’t know if we were saying something wrong, and we wouldn’t know what to talk about … you don’t know if they (residents) like to talk about something personal, and if we dig too deep…. I am just here as an NA, so it would seem to be not that good to know too much about their personal issues…. Since we are not that clear about their conditions, it could be a big problem if we say something upsetting by mistake.” (NA 3)	Barriers to closer interactions	“It is not the NAs’ responsibility … to relieve your emotions…. What they are responsible for taking care of the residents’ physical health…. The NAs are too busy to listen to you…. I think it is beyond their responsibilities…. They are not supposed to deal with your inner feelings.” (RE 14)
			“Feeling unhappy is my own problem and shouldn’t be shared with NAs … to bother them because, firstly, they have no time and, secondly, it is inappropriate to bother them (NAs) as they have no responsibility for your personal problems.” (RE5)
Second type: Cordial interactions intended to maintain a harmonious atmosphere			
Remaining emotionally detached from residents	“We talk to them in a friendly manner … usually about general topics…. If we were really close, like family (with residents), I think it would make my work tougher. What if they (residents) pass away and I can’t get over it. There are just so many dramatic changes for residents….” (NA 10)	Feeling secure by keeping a distance from NAs	“I seldom chat with the NAs … other than daily greetings. We should be careful about what we say, right? People shouldn’t gossip…. NAs work here, so we shouldn’t talk about anything in depth, right? … to them or to anyone else…. There are no deep relationships here, and it is very hard to get to know each other well…. My need? Ha, ha, I am magnanimous. I don’t think about anger or happiness…. I don’t need to think about anything, just eat whatever I have, sleep whenever I can.” (RE 15)
Refusal by residents to interact socially	“The residents don’t think about building closer relationships with us. They just treat us as workers who offer personal care.… I think that they do not think much about our relationship. I respect their choice to maintain a formal care relationship….” (NA 10)		“I rarely share my personal issues with NAs. We have different thoughts and are not close … it is so hard to be close with each other (NAs)…. Sometimes, … I prefer to share my feelings, my issues, with my kids.” (RE 7)
	“Some residents responded coldly to us. We would just smile and walk away…. We learn to bear it; we don’t force them, so as to avoid conflict.” (NA 12)		
Third type: Reciprocal social interactions intended to develop closer rapport			
Using humor on the residents	“When I saw this old lady, I’d yell at her. And she would probably yell back at me since she knew I was just kidding…. It is just a kind of communication, which may be misunderstood by others, but we were just having fun…. So I could joke with those who were up for it, or just say ‘hi’ to those who disliked joking. We got to know the characteristics of the residents after working here for a while.” (NA 8)	Accepting kidding for the purpose of being sociable	“Sometimes the NAs would joke with me … saying that I am pudgy but have nice skin.… Or they would say things like, ‘You are so pretty, I want you to be my mom.’ So I responded, ‘Then I’ll call you “daughter”.’ … But some residents won’t accept being called a ‘fat lady.’ … If you understand that it is a joke, you will feel okay. So NAs really need to know the residents, in order to know what is acceptable for them (the residents), as it is not okay to say this kind of thing to everyone.” (RE 4)
Receiving attentive concern from residents	“I sometimes had a sudden headache and would say to myself: ‘Ouch! Such a bad headache, so annoying! I should take a Panadol.’ The resident heard that and offered me some herbal massage ointment … which means that the resident also pays attention to us. [Interviewer: How did you feel about it?] Very happy. That resident was also happy when I accepted her offer and asked me later about whether or not my headache had been relieved…. Because this kind of interaction made her (the resident) feel happy, she would continue to care about us (NAs).” (NA 12)	Showing care and concern to the carers	“An NA didn’t feel well, I attempted to comfort her … and I would also ask her if she was feeling better the next day.… We know, you (NA) care about me, and so do I…. She would ask me things like “Do you want some more food? Why are you eating so little?” (RE12)
			“Mostly I ask NAs first, things like, “How are you doing today? Tired?” … If you actually worked in a nursing home, you would know that some residents are tough to handle.… I treat the NAs like my friends, which means that we share what has happened here and comfort each other.” (RE 9)
Establishing good relationships through sociable contacts	“Some residents remembered that I had a grandson, and would ask me to show them (residents) his photos (the NA’s grandson)…. They praised my grandson as being very cute and chubby.… Some of them would then show me the photos of their grandchildren…. This was how we started to build up a nice relationship….” (NA1)	Enhancing individuality by being addressed using nicknames or family metaphors	“I really love to hear the NAs calling me ‘teacher’ … because I was a teacher for my whole career.… [Interviewer: How do you feel when the NAs call you that?] Fabulous! … Because at least they know I was a teacher, not someone ordinary, but an intellectual…. The way they call me that, it feels like they are showing respect.” (RE 13)
	“Some residents, who are more open, love to share their past with you.… They are happy and even more relaxed when you chat with them as friends. If you only behave like a formal care provider, they won’t talk that much. So it is important for us to be open with them, then they feel much better!” (NA7)		“My surname is same as two NAs…. So they usually call me ‘big sister’ and I call them ‘little sister.’ … I feel happy we call each other ‘sister’ … it seems that they all familiar with me and treat me well.” (RE 6)
Being sensitive to the residents’ need for emotional support	“I saw that the resident couldn’t stop crying in her bed when I was walking around the room, so I asked what had happened.… She told me something about her family history.… I comforted her patiently … she felt a lot better.… Since then, she always tell me that I am the one who really cares about her (the resident).… She gave me fruit occasionally.… This showed me that if you care about the residents, they appreciate that.” (NA 9)	Offering help to NAs to show my appreciation	“You shouldn’t mess things up for the NAs to clean up, right? When I am dining, I pick up the rice myself if it accidentally falls on the floor…. They praise me, ‘Wow, you made it so clean!’ When I see that they are doing things that I can do, I think why not just do it myself? They are working the whole day without breaks. It is not easy.” (RE 12)
		Respecting my individuality and preferences	“I feel that the NAs care about me.… Sometimes I say I’d love to have some ginger…. I get a small pack of ginger the next day from the NAs…. They know what I like to eat…. Because they (the NAs) see what I eat daily…. Also, I have to eat something before I go to sleep because our dinner is early. So I eat bread, biscuits, etc. Since they (NA) know this, they always drop some food off for me.… In return, sometimes my friends come and visit me with fruit or biscuits, etc., and I would give the NAs some….” (RE 1)

### Physiologically-oriented daily interactions

The interactions between NAs and residents most frequently mentioned by both groups were those that occurred during the delivery of care. Participants from both groups perceived that the primary responsibility of NAs is to fulfill the physiological needs of residents. Thus, both groups described many scenarios that occurred when the NAs were providing nursing care to residents. Those interactions were driven by the residents’ physiological needs. Some NAs saw themselves as being at the bottom of the nursing home hierarchy with their primary duty of focusing on the residents’ personal care. Thus, their formal interactions with residents were mainly related to the residents’ health issues. Besides attending to the physiological needs of the residents, some participants in the NA group felt that providing support to meet other aspects of the residents’ needs exceeded the boundaries of their role. Theme I identified from the NA group describes how NAs saw their primary caregiver role as determining their daily interactions with the residents.

#### Worrying about going beyond the boundaries of an NA’s responsibilities (NA group)

The majority of NAs perceived themselves as incompetent or irrelevant when it came to handling matters beyond physical health during their daily interactions with residents. They believed that such matters were not among the duties of their job and might not be supported by their supervisors. They believed that providing psychosocial support required the residents to disclose personal issues, and that this kind of support would be extended after they developed a closer relationship with the residents. They believed that this was not compatible with their major duty in a nursing home.

##### Falling under the responsibility of other professionals

Many NAs mentioned that they were not health professionals when compared with nurses, social workers, or physiotherapists. When they observed emotional distress in the residents, they perceived that their major role was only to report this to other professionals. They believed that, based on their training, they could provide nothing of significance to fulfill the psychosocial needs of the residents. Developing a closer relationship with residents should not be their top priority, according to the duties of their job in the nursing home.

##### Perception of incompetence and irrelevance

Some NA participants recalled that some residents who had limited family support were prone to exhibit signs of depression, such as crying or social withdrawal. In such cases, they would be very careful when providing care to those residents, so as to avoid touching on their personal and family problems. According to the NAs, this was the most effective way of preventing themselves from inadvertently provoking negative emotions in the residents. They explained that this went beyond the duties of their job. They also had no confidence and received no training in handling negative emotions among the residents. Also, some NAs considered it more appropriate for them to focus primarily on the residents’ physical health. Little interest was shown by some NAs concerning issues other than the physical needs of their residents.

##### Boundaries inherent in the nature of an NA’s role as a formal physical caregiver (Resident group)

The resident group also regarded interactions between NAs and residents that are driven by health needs as being the norm. Many residents stated that they understood an NA’s primary role as being that of a formal caregiver, with responsibility for offering direct personal care to residents. On the other hand, residents were care recipients in need of assistance due to their impaired physical / cognitive functions. Some residents considered the formal caregiver-care recipient relationship that drove their major daily interactions with the NAs as being physiologically-oriented. They considered that the NAs largely focused on their physical health needs. Therefore, they seldom expected to receive support in other aspects from the NAs. The residents’ daily experiences of interactions with NAs reinforced their perceptions of NAs as formal caregivers who primarily address the physical needs of residents. They revealed that they rarely think of establishing stronger rapport with the NAs.

##### Driven by the residents’ demands relating to their physiological needs

Some residents mentioned that the division of labor in a nursing home was like having a guideline for them to understand the responsibilities of different workers. They showed that they understood that an NA’s primary responsibility is to offer direct personal care. Therefore, they considered it inappropriate to demand that NAs provide support beyond that of attending to their physical needs, as this went beyond the duties of their job. In addition, some independent participants perceived that talking to NAs meant seeking their assistance in personal self-care activities. They saw it as unnecessary to have such a connection with NAs when they are able to take care of themselves.

##### Barriers to closer interactions

Although a few residents expressed the need to have someone in the nursing home comfort them when they were sad, the NAs’ busy routine made these residents hesitant about expressing their negative emotions to the NAs. They usually suppressed their true feelings during their daily interactions with the NAs. Some residents also believed that it was difficult to establish rapport with NAs as their daily contacts with them were mainly physiologically-oriented. This reduced their motivation to establish closer interactions with or to seek support from NAs. Several residents emphasized that, because the NAs were busy with their work, they had limited contact with the NAs during the latter’s leisure time. Thus, the interactions between the two parties took place predominantly during nursing procedures. Apart from these interactions, encounters with NAs were restricted to a few words of greeting.

Overall, there was physiologically-oriented interaction focusing on physical health needs with little psychosocial/emotional involvement. The perception was that NAs were not trained and had no role to play in the provision of psychosocial support, which hindered the NAs’ intention to establish rapport with the residents. This perception was reflected in their daily interactive behaviors with residents. Eventually, this situation may reduce opportunities for the residents to express their personal concern and gain support from NAs, despite the fact that NAs provide most of the personal care to residents. Also, it does not seem to be easy to develop rapport because of the boundaries between both parties inherent in the formal caring relationship.

### Cordial interactions intended to maintain a harmonious atmosphere

“Cordial” describes relations that are friendly but formal and polite. This was another common interaction described by participants in both groups. Maintaining a harmonious atmosphere during daily interactions was perceived to be important by the participants. Some NA participants emphasized the importance of maintaining a good but formal caregiver-care recipient relationship, as they believed this was an effective way of ensuring the quality of the care that they provided. Similarly, many resident participants believed that they should be polite to NAs to avoid conflicts and ensure harmony in their daily interactions. Thus, both groups described many of their interactions as rather shallow. As NA participant said, *“Of course, we greet each other when we meet in the morning … maybe have some social small talk … such as about the weather … or about what has happened recently in the nursing home…. We don’t talk about things other than our work with the residents….” (NA15).* Likewise*,* many residents saw NAs as having a tough job and showed their respect for them by greeting them courteously when they met. Some residents stated that they seldom contacted NAs as they could take care of things on their own. They saw this as reducing the workload of the NAs and as an alternative way of showing their respect for them. They felt that this approach could promote a harmonious relationship with NAs. Thus, the daily contacts between these two parties were friendly and polite, but formal and superficial.

#### Remaining emotionally detached from residents (NA group)

Some participants expressed the worry that by building close relationships with the residents, they ran the risk of arousing negative emotions or feelings attributable to the residents’ unstable health conditions. This could then interfere with their duty to provide care to the residents and destroy the existing harmonious caregiver-care recipient relationship. Therefore, they considered it inappropriate to extend NA-resident relations beyond a formal caring relationship and preferred to maintain a distance in their relationship with residents.

##### Refusal by residents to interact socially

There were occasions when some residents were introverted or maintained their distance from NAs. According to some NA participants, those residents usually responded coldly to NAs. Despite the residents’ lack of interest in interacting more closely with them, the NAs claimed that they accepted such behavior in order to maintain harmony. They believed that this also allowed the residents to perceive that they were being cared for. A participant in the NA group pointed out that she understood the residents’ rationale for responding in such an unsociable manner. However, it was difficult for NAs to have closer interactions with such residents.

#### Feeling secure by keeping a distance from NAs (Resident group)

Generally, most residents were satisfied with their cordial contacts with NAs and considered the small talk that they engaged in as demonstrating harmony and respect. A few residents regarded nursing homes as hotbeds of gossip. They felt secure and comfortable about keeping a distance from others and saw it as a protective mechanism to avoid becoming embroiled in negative talk. They considered gossip as bothersome and malicious, and possibly leading to disharmony among the people around them, including the NAs. This was consistent with the residents’ feeling of security and comfort with just having a nodding acquaintance with the NAs. Some residents stated that they preferred to vent their grievances and seek emotional support from their family while the latter were visiting them. They felt safer about sharing their inner feelings with people with strong bonds with them, such as family members or old friends.

Maintaining a polite but distant formal care relationship was perceived by both parties as an effective way of maintaining a harmonious atmosphere in nursing homes. Thus, their interactions predominantly consisted of greetings, social small talk, or general and shallow contacts, with little mention of the residents’ emotional/psychosocial needs. The intention of both parties to develop a stronger connection was modest.

### Reciprocal social interactions intended to develop closer rapport

Some participants in both parties emphasized that the intention behind some of their daily interactions with residents / NAs was to establish a friendship between them. Although most of the interactions that they described consisted of social chitchat with limited in-depth sharing of feelings, the participants explained that this was how they showed their understanding and mutual concern. They perceived that the aim of these actions was to establish a better relationship with each another. “Mutual concern” refers to reciprocal relationships in which two people develop a mutual understanding and know how to get along with one another [17]. Interestingly, similar themes were identified from both groups that echo each another on the support for this type of interaction. Both parties expressed concern and care to reflect understanding towards their counterparts. The NAs’ care was based on their appreciation of the residents’ unique identity and their recognition of the residents’ individual needs, whereas the residents showed their understanding, attention, and concern about the pressure faced by the NAs and their heavy workload. The residents considered that both parties contributed actively in building a friendship between them. As the NAs’ caring actions and respect for the individuality of the residents were acknowledged by the residents, both parties gradually developed closer rapport.

#### Using humor on the residents (NAs)

The NAs illustrated their understanding of the residents’ individuality with examples of how they used humor to show friendship to particular residents. They claimed that they adjusted the content and tone of conversations in accordance with the residents’ reactions and preferences. They highlighted the importance of understanding the residents’ personalities and level of acceptance, and the topics that they liked to talk about, so that the NAs knew which residents accepted their humor. They showed themselves to be alert in avoiding triggering negative emotions in the residents. The NAs believed that residents who responded positively would understand the NAs’ intentions and accept this kind of communication. From the NAs’ point of view, free expression during joking reflected understanding and a friendly relationship between themselves and the residents.

#### Receiving attentive concern from residents (NAs)

As formal caregivers in a nursing home, NAs often expressed their concern about the residents’ health and provided care or advice to them. When in return the residents expressed verbal concern about the NAs’ health or daily life, the NAs saw these expressions as a form of attentiveness and interest in establishing a closer relationship. The NAs also saw this attentive concern from residents as indicating that they were important to the residents. The NAs responded positively to the residents’ contributions to developing a good relationship of mutual concern. For example, one NA recalled an occasion when a resident was aware of her headache and offered her some herbal massage ointment. The NA was surprised that the resident had noticed her headache. She considered that the resident’s action reflected attention and concern, which gave her a sense of warmth and of being cared for by the resident. Also, the NA perceived that her acceptance of that resident’s offer indicated appreciation and signaled that she welcomed this social interaction.

#### Establishing good relationships through sociable contacts (NAs)

Some NAs mentioned having free and social interactions with residents who were relatively open and willing to establish a closer relationship. Both parties initiated the interaction with self-disclosures or by showing interest in their counterpart. Diverse topics in the conversations / interactions were described by the NAs. They considered this kind of interaction as indicating that both parties treated each other as friends instead of merely as caregiver and care recipient. Sometimes, NAs shared information about their family and hobbies with the residents. This was to let the residents perceive their closeness.

#### Being sensitive to the residents’ need for emotional support (NAs)

Some NAs showed a comparatively high level of awareness about the residents’ psychosocial needs and considered these to be as important for the residents’ health as physical support. Some NAs pointed out that it was common in a nursing home to encounter emotional distress among the residents. In such cases, they would pay attention to the residents’ mood and do their best to help the residents calm down. An NA described an experience of noticing and relieving the emotional discomfort of a resident. Later, the resident showed her appreciation to that NA, indicating that the resident knew that she was being cared for. This incident strengthened the value that this NA placed on providing psychosocial and emotional support to residents.

#### Accepting kidding for the purpose of being sociable (Resident group)

Some residents mentioned that they had a good sociable relationship with the NAs who provide them with daily personal care. They highlighted the free and casual expressions that they used with the NAs, such as joking and kidding, indicating that their formal caring relationship extended somewhat to friendship. Residents considered joking and kidding with NAs as pleasant interactions in which both parties felt free to express and show interest in each other. They perceived that the NAs understood what the residents liked and disliked and would adjust the conversation according to the residents’ preferences. The residents stated that they responded to the jokes in a manner similar to that of the NAs to show their acceptance of the initiative to shift from a formal towards a more casual relationship. According to McCabe’s study [27], relationships that are characterized by friendliness and humor enhance the self-esteem and happiness of patients, and allowing free expressions makes the interactions between caregivers and care recipients more open and casual.

#### Showing care and concern to the carers (Resident group)

Some residents highlighted the understanding and natural concerns that characterized their interactions with NAs, and perceived that their relationship was like that between friends. A resident described how she showed her concern toward an NA who was ill, and perceived that this was a manner of expressing reciprocal care in their daily interactions. That resident treasured this kind of reciprocal care, which made her feel that both parties were showing each other affection and care. The residents regarded their relationship as a friendship in which both parties paid attention to and valued each other.

#### Enhancing individuality by being addressed using nicknames or family metaphors (Resident group)

The residents saw the fact that they were addressed by NAs using nicknames or family metaphors as indicating that they were accepted as unique members in the nursing home. The residents developed feelings of familiarity and connection with the NAs due to an increased sense of belonging and acceptance arising from this simple daily address by the NAs. The residents associated this form of address with the recognition of their own individuality and self-identify. A male participant mentioned having been a teacher for many years before retirement. He perceived that being called “teacher” by the NAs indicated the NAs’ recognition of his good education. He claimed that although his physical health was gradually deteriorating, this title reinforced his identify and improved his self-esteem. Similarly, another woman in the resident group mentioned that being called “Big sister” by NAs who shared her surname gave her a sense of familiarity and connection with the NAs.

#### Offering help to NAs to show my appreciation (Resident group)

Some residents shared their experiences of showing care and support to NAs by actively helping the NAs with their tasks. Their impression was that the NAs were extremely busy in caring for the residents; therefore, they considered their help as representing collaboration, appreciation, and understanding of the NAs. When NAs responded with compliments, that made the residents feel as if they were working together to build a closer relationship and being recognized as members of the community.

#### Respecting my individuality and preferences (Resident group)

Residents felt that they were being cared for when their individual needs, preferences, and habits were recognized by NAs. Several residents were impressed and felt a sense of warmth when the NAs remembered their individual preferences and needs. A female resident shared her experiences of frequently receiving food that she liked from NAs. She considered that the NAs were doing this favor particularly for her, implying a higher level of rapport and value between them. It also represented the NAs’ concern for and recognition of her individual preferences and needs. Being fulfilled on a personal basis led to a sense of security. Upon receiving food from the NAs, she reciprocated to show that she treasured and valued this kind of interaction.

In this type of interaction, both parties expressed their intention to establish a closer rapport with their counterpart by doing favors for each other. The NA’s commitment to respecting the residents’ individuality through different daily interactions strengthened the residents’ sense of togetherness and security in extending the formal care relationship to a closer friendship with the NAs. The residents responded by showing their willingness to cultivate stronger connections with the NAs. This promoted the building of trust and attachment between the two parties, which led to the extension of more social support and the recognition of individual needs beyond those involving physical health.

## Discussion

This study contributes qualitative data by identifying the perspectives of NAs and residents on their daily interactions. Three types of interactions were found, namely: 1) superficial physiologically-oriented interactions, 2) cordial interactions, and 3) reciprocal social interactions. In addition, our findings showed the participants’ intentions and beliefs in relation to different interactions, and how these intentions and beliefs direct both NAs and residents to exhibit different interactive behaviors. After understanding that the common interactions in nursing homes are affected by the NAs’ and residents’ beliefs and intentions, focused strategies can be designed to support the establishment of more rapport between NAs and residents.

The perceptions held by NAs and residents of the role of the NA were found to be crucial in influencing their interactive behaviors. NAs were viewed as formal caregivers who focus on the physical needs of residents, while providing residents with psychosocial or emotional support was considered the responsibility of other health professionals (such as social workers). Some residents had low expectations of support from NAs beyond the provision of basic personal care, while some NAs preferred to only report to other health professionals when encountering needs of residents beyond those of personal care. Subordinate to professional health caregivers, NAs work under the close supervision of professional staff to provide basic personal care to residents [7] The hierarchical division of labor in the nursing homes strengthened the NAs’ role as hands-on workers. Rigid role restrictions suppressed their preference for being open with residents [28]. In addition, the training provided to NAs only targeted physical care, which also influenced how NAs prioritized their work. Some NAs considered it inappropriate to develop rapport with residents. Some even believed that NAs could not provide any emotional support to residents. This perspective may be due to the fact that the NAs received very little training on promoting interpersonal relationships or caring for the psychosocial needs of residents. Many NAs expressed worries about provoking adverse reactions due to their lack of knowledge in handling the negative emotions of residents. These worries reduced their motivation and confidence in developing better interpersonal relationships with residents.

Due to their heavy workload, the NAs usually interacted with residents in a cordial manner. Their intention was to deliver nursing care to residents in an efficient manner. They prioritized the care based on urgency. Thus, nursing staff pay more attention to the residents’ physical health than to aspects considered less urgent, such as the residents’ emotional or psychosocial health [29]. The participants in the resident group described the NAs as “non-stop workers.” They stated that the NAs concentrated on their physical care needs and rarely initiated any gesture to develop a closer relationship with them. The residents considered it rational for NAs to concentrate on their caring tasks and regarded approaching NAs for support beyond that for their physical health as interference. The residents’ daily experiences reinforced the view that their interactions with NAs are physiologically oriented. In fact, all of these experiences rationalized the superficial and task-oriented interactions between NAs and residents. Participants in both groups generally accepted this task-oriented interaction, although previous studies have shown that such superficial interactions often caused residents to feel detached, ignorant [6, 30], and inhuman [31].

Participants from both groups also thought that there were many uncertainties in the social networks built in nursing homes. Relationships could end suddenly due to the unstable condition of the residents’ health or to the resignation of caregivers, which would cause grief and loss. Some participants said that they preferred to keep a distance in their relationships, believing that this is an effective way of maintaining harmonious relationships with others. Harmony was emphasized as a way of ensuring a peaceful life in the nursing home and good care. The participants felt that getting closer with people in the nursing home would lead to the risk of becoming embroiled in gossip and conflicts. Therefore, some residents said that they rarely disclosed personal issues to people in the nursing home, including NAs, and only engaged in greetings and social small talk.

A sense of kinship has a powerful influence on the relationships of Chinese people [32]. It affects Chinese people’s expectations of the closeness of their relationships with others as well as their behaviors when they interact with people outside the family. Under this influence of traditional Chinese culture, many participants in the resident group preferred to obtain emotional support from their families. They perceived psychosocial and emotional support as something that should come mainly from family members. Although visits by family members were infrequent, some residents wanted to wait for the next visit from their family to share their personal feelings with them. In addition, older people usually prefer to interact with people from their old social networks. Some residents said that it was difficult to start other close relationships like the ones they had with family members or old friends. They generally regarded the nursing home as just a place to live, where the relationships with people are shallow, the lifestyle highly structured, and the activities restricted. As strong family bonds are valued in Chinese culture, this may have lowered the residents’ expectations of getting closer to other people. This is another possible explanation for the residents’ acceptance of shallow and task-oriented interactions with NAs.

Although residents preferred to get their emotional support from family members, because visits from family were often infrequent, most residents had weak social networks. When they were distressed, some residents tried to suppress their emotions and rarely sought help from others. Although residents saw social workers as a source of psychosocial and emotional support, they seldom actively sought help from them due to the shortage of manpower. Residents generally felt that there was a lack of opportunity to share or seek emotional support when needed. This threatened the residents’ psychosocial wellbeing, especially when they needed comfort for their worries or negative emotions. A lack of companionship exacerbated feelings of loneliness among the residents. In contrast, it was found that having relationships to trust with caregivers helped the residents to express themselves more openly, thus facilitating the provision of social and emotional support [33, 34]. This helped to build feelings of reassurance and security, as the residents knew that their needs would be addressed by the caregivers.

NAs represent the taskforce that provides the most direct personal care to residents in nursing homes. This gives NAs the advantage in building rapport and supporting the residents’ psychosocial wellbeing. Although their obvious physical caregiver role has a dominant influence on daily interactions between NAs and residents, some NA participants considered physical and psychosocial care to be equally important in promoting a sense of wellbeing in residents. They were motivated to develop more rapport with residents. Some participants in the NA group had discussed the strategies that they used to get along with residents during their daily contact, such as kidding to show harmony, doing favors to represent familiarity, and recognizing a resident’s individuality to show respect. The residents’ motivations for becoming involved in closer NA-resident interactions were influenced by the NAs’ intention to develop closer rapport. During the interviews, some residents did express a need for psychosocial or emotional support. However they rarely confided this need to the NAs, unless they sensed that a particular NA cared for them as individuals and initiated more personal interactions [33]. The NAs’ initiative in getting to know the residents as individuals was like a sign permitting deeper interaction and allowing room for the residents to express their authentic selves. Similarly, the positive response of the residents to the NAs’ friendly approaches encouraged the NAs to establish a closer relationship with them.

The findings of the study reflected an incremental improvement in rapport between the NAs and the residents. From a purely physiological orientation, the NAs strove to express cordiality with the aim of achieving harmony, and encouraged the residents to reciprocate, showing their desire to achieve closer relations and greater fulfillment by promoting a sense of belonging, individuality, and psychosocial support. To enhance the wellbeing of residents, their physio-psychosocial needs must be satisfied. The residents’ basic physical health needs must be met before they and their caregivers can step forward to establish rapport and become more socially engaged. Although all health professionals working in nursing homes have the responsibility of addressing the residents’ needs in all aspects, the NAs are also in a good position to provide instant support to the residents, as they have numerous opportunities for coming into close contact with them. Therefore, the building of good rapport between the two parties is one more way for residents to obtain support. This enriches the residents’ social support in nursing homes. Although some participants in the NA group were found to have established good relationships with residents and developed affective communication skills, it is believed this was a result of their natural talent, rather than of their training [14]. Rather, the NAs’ perception of their interpersonal skills as insufficient caused them to feel stressed out and to lack confidence during their social interactions with residents [35]. Thus, some NA participants still believed that developing rapport with residents went beyond the scope of their duties. To improve NA-resident interactions, it is pivotal to provide organizational support such as by modifying training and practice guidelines, and providing NAs with accessible professional support. Aside from the physical aspect, it is essential to train NAs to be more aware of the importance of providing psychosocial/emotional support and building rapport on top of providing personal care. Also, NAs need to be trained in how to integrate social interactions into daily care if they are to become competent and confident about promoting a sense of wellbeing in the residents. Creating platforms for social interaction between NAs and residents, apart from the time when the NAs are engaged in delivering care, will provide more opportunities for the kind of daily contact that facilitates the building of good relationships [36]. Finally, adequate staffing is crucial, in order to prevent NAs from becoming overloaded with nursing tasks.

### Limitations and suggestions for further study

The findings of the study were based only on the participants’ own perspectives, and no triangulation was done to verify the collected data. The participants might have selected their more impressive experiences of harmony and good support to show that they are good caregivers or cooperative residents. They might have been providing pleasant answers that favored the interviewer’s perceptions of good NA-resident interactions. However, if this was the case, this did not prevent the NAs from describing task-oriented interactions, which, from the perspective of some NAs, also reflected good care. Likewise, the residents also described some negative experiences related to their daily interactions with NAs. Strategies were used in this study to address this issue, including giving all of the participants a detailed explanation of the purpose of this study prior to the interviews, and emphasizing that their identities and the data that they provided would remain confidential. Throughout the data collection process, the researcher was also alert to personal preconceptions about positive interactions in order to minimize her influence on the participants’ sharing of their views. Including observation as a part of the data collection method can improve the credibility of the findings. It is suggested that, in a future study, triangulation of data using individual interviews and observations be conducted to provide a more thorough reflection of the daily interactions between NAs and residents.

This study provides a basis for understanding interactions between NAs and frail elderly people. Other stakeholders such as nurses and the home manager also play a crucial role in building NA roles that influence NA-resident interactions at an organizational level. Therefore, in future studies it is worth investigating stakeholders’ views on the role of the NA, as well as their expectations of NA-resident interactions. In addition, it was pointed out in this study that Chinese cultural values on close family bonding allowed the residents to perceive that it was hard to establish warm and trusting relationships after moving to a nursing home. This lowered their expectations and dampened their intention of getting closer to others; thus, it became easier for them to accept distant relationships with NAs. The majority of studies investigating interactions between nursing staff and residents were conducted in Western countries; therefore, it is worth learning more about how the Chinese culture facilitates or impedes NA-resident interactions, and the corresponding impact on the psychosocial wellbeing of the residents. Furthermore, there is still little knowledge about the education of NAs in relation to building rapport and providing social support. Previous studies mainly targeted the training of nurses and other professionals. More effort should be put into examining strategies specifically designed to enable NAs to establish good relationships with and provide social support to residents.

## Conclusion

By illustrating how the underlying intentions and beliefs of NAs and residents influence their daily interactions in nursing homes, this study contributes valuable information on how to identify potential strategies to encourage the development of rapport between NAs and residents. The findings revealed that an over-emphasis on the formal caring relationship and excessive concern about maintaining a harmonious atmosphere partly contributed to the existence of superficial and distant relationships between NAs and residents. Building close rapport through repeated, reciprocal, social interactions takes time. The findings in this study show if NAs and residents have the good intention of establishing closer rapport, the two parties will do favors for each other by using humor, showing care and concern, engaging in social chitchat, and respecting each other’s individuality. It would seem that all of these favors can be easily integrated in the NAs’ daily interaction with residents while providing personal care. It is important to raise the NAs’ awareness of the importance of building good relationships with residents and their competence in doing so. Here, modifying the NAs’ training and establishing institutional policies are crucial steps. Positive interaction not only improves the psychosocial wellbeing of the residents but also encourages them to cooperate and participate during the delivery of care, in turn promoting their overall health and contributing to the NAs’ job satisfaction. Thus, good NA-resident interactions offer residents one more platform for gaining social support and building good rapport in nursing homes, and should be encouraged.

## Abbreviations

NAs: Nursing assistants
CMMSE: Cantonese Mini-Mental State Examination
